# Local Analgesia in Surgical Operations

**Published:** 1914-09

**Authors:** A. L. Flemming

**Affiliations:** Senior Anæsthetist to the Bristol Royal Infirmary


The Bristol

Medico-Chirurgical Journal.

*"Scire est nescire, nisi id me*
*Scire alius sciret."*

SEPTEMBER, 1914.

### LOCAL ANALGESIA IN SURGICAL OPERATIONS.

BY

A. L. FLEMMING, M.B., Ch.B. Bristol,

*Senior Anæsthetist to the Bristol Royal Infirmary.*

THE skilful employment of local analgesia in surgery can only be relied upon where due consideration is paid to a variety of factors, so numerous that their mastery calls for no small degree of patience, and so essential that the neglect of any one of them will almost certainly lead to some degree of failure.

The factors which it is necessary to consider may be reviewed under the following headings :—

1. The type of patient.
2. The nature of operation.
3. The selection of drug.
4. The method by which the drug is administered
5. The technique employed.

1. *The type of patient.*

Local analgesia may be used with confidence in adults of good physique, especially if possessed of a calm disposition, and if showing a capacity for tolerating a certain degree of discomfort without becoming apprehensive or frightened, but in the case of nervous and irritable patients it should generally be avoided.

In some instances, however, where an insuperable dread of a general anæsthetic has become established local analgesia offers a most efficient substitute.

Children under twelve years of age are not good subjects, owing to their susceptibility to toxic symptoms and to their want of control. If however, great care is exercised in the dose to which they are submitted, children as young as five or six may be successfully operated on under this method. Struthers and others have reported many cases.

Old patients, and those who have become weakened by chronic disease or septic processes, are prone to develop symptoms of poisoning if the quantity of drug used is not smaller than the average dose, and they are apt to suffer from shock if the quantity has not proved sufficiently powerful to render the operation painless. This class of patients, therefore, requires careful handling.

Some individuals undoubtedly show an idiosyncrasy for the poisonous action of cocaine, eucaine, and novocain, but with modern methods and suitable precautions even this danger has become almost negligible.

2. *The nature of operation.*

So much depends upon the circumstances surrounding each case, that no general law can be laid down as to which particular operations ought to be performed under a local and which under a general anæsthetic.

The work of such men as Schleich, Cushing, Braun, Reclus and others has demonstrated that practically any operation may be performed under local analgesia, a fact of special interest to the surgeon who meets with the increasingly rare contingency of having to work without skilled assistance or the ordinary facilities to which he is accustomed.

When a table is made of cases where a general anæsthetic would jeopardise life, and where analgesia is therefore the method of election, the list is a limited one, and includes such operations as tracheotomy for acute or increasing respiratory distress, especially if accompanied by cardiac failure or grave toxæmia, as in diphtheria; enterostomy or herniotomy for intestinal obstruction with fæcal vomiting; cystotomy for prostatectomy when complicated by the presence of bronchitis; exploratory incisions in cases of advanced malignant disease of the abdomen; gastrostomy in senile subjects in advanced stages of inanition; superficial operations on subjects who suggest a tendency to cerebral hemorrhage, or who are suffering from such diseases as diabetes or exophthalmic goître.

In all other instances the preference for local over general anæsthesia will be a question of convenience rather than one of safety, and will apply to cases too trivial to justify the inconvenience of a general anæsthetic; instances where the surgeon is single-handed or where the wish or fears of the patient demand local analgesia.

3. *The selection of drug.*

Cocaine when first introduced was used in unnecessarily concentrated solutions such as 3, or 5, or even 10 per cent., instead of ½ or 1 per cent., and frequent instances of poisoning resulted. It was pointed out by Schleich that efficient analgesia could be obtained with such weak solutions as ½ or 1 per cent., also that when used in these dilute strengths a larger total dose of the drug could be tolerated with impunity. The experimental and clinical results of Braun showed that whereas $\frac{3}{4}$ grain of cocaine represented the safety limit when used in a 1 per cent. solution, $1\frac{1}{2}$ grains could be given in safety in $\frac{1}{10}$ per cent. solution. But $\beta$-eucaine made its appearance with claims to a lower degree of toxicity, and to a large extent displaced cocaine in this country. The safety limit of this drug when combined with adrenalin was found to be 2 grains when used in a $\frac{1}{2}$ per cent. solution, and 6 grains when used in a $\frac{1}{10}$ per cent. solution. $\beta$-eucaine thus gained favour as being comparatively safe, but it brought discredit to the method owing to its lack of diffusibility and the consequent uncertainty of the resulting analgesia.

Latterly novocain and quinine-urea hydrochloride have taken the field almost exclusively in local infiltration and nerve-blocking methods.

Novocain gives reliable analgesia in $\frac{1}{2}$ to 2 per cent. strengths, but its effect is more transient than that of cocaine—15 minutes compared with 30 minutes (approximately); its toxicity stands to that of cocaine as 1 is to 7; its maximum dose is 7 grains, and it has the advantage of being sterilizable by boiling.

Urea quinine hydrochloride has a very slight degree of general toxicity, but it produces a peculiar local reaction in the tissues with which it comes into contact. The drug will undoubtedly grow in popularity when the local redness and induration which sometimes follow its use are better understood. These local symptoms may last for some days, but they may, in a large measure, be avoided by the employment of weak solutions, 1 per cent. never being exceeded, and by injecting only into healthy tissues.

The drug is not destroyed by boiling, and the analgesia it produces lasts for a period varying from one hour to twenty-four hours.

4. *The method by which the drug is administered* has a direct bearing upon the selection of drug to be used.

For topical application, as obtains in nasal surgery, cocaine is most suitable owing to its diffusibility. On the other hand, urea-quinine hydrochloride in strengths of 10 or 20 per cent. may be used (Hertzler), but has no blanching action upon the mucous membrane. The urea-quinine salt may be used freely to analgesise the mucous membrane of the bladder.

For œdematisation or infiltration of large areas of connective tissue novocain should be used, and the urea-quinine salt should be avoided, as it produces too much lymph exudation.

Where large areas have to be rendered analgesic it is wise to employ the method of nerve-blocking by injecting novocain or urea quinine, either around the nerve tissues concerned or into the sheath of each nerve after exposure by dissection.

When employing urea quinine for perineural injection, it must be borne in mind that the action of the drug may be delayed for ten minutes or even longer.

5.—*The technique employed.*

The success of the method depends largely upon perfection of technique, which must include—gentleness in manipulation, avoidance of dragging on or of stretching tissues, a full appreciation of the relative sensitiveness of various anatomical structures, and the employment of really sharp cutting instruments and of forceps as light as may be compatible with good workmanship. Moreover, all concerned must be willing, on the slightest evidence of pain, to wait for further injection or for the development of analgesia.

*In conclusion.*

Local analgesia has been accredited with a capacity for preventing shock far exceeding that possessed by general anæsthesia.

In this connection it must be remembered that with skilfully-administered general anæsthesia shock is not met with so frequently as some of the advocates of local analgesia would have one to suppose. Moreover, in many of the operations where anoci-association is imperfectly practised brilliant results are claimed. The strength of novocain solution generally used is $\frac{1}{4}$ per cent., and if this strength is used without a general anæsthetic it will be found to give a very deficient analgesia. Moreover, clinical experience and recent experimental work seem to point to the fact that general anæsthesia has the property of preventing the occurrence of shock.[1]

[1] Dr. Mann, *Johns Hopkins Hosp. Bull.*, 1914, xxv. 205.

